# Supplementary material for: Exploring the behavioral determinants of COVID-19 vaccine acceptance among an urban population in Bangladesh: Implications for behavior change interventions
Source: PLoS One. 2021 Aug 23;16(8):e0256496. doi: 10.1371/journal.pone.0256496 (PMC8382171; doi:10.1371/journal.pone.0256496)
Supplement: S3 File — (PDF) [file pone.0256496.s003.pdf]

বাংলাদেশে ঢাকা শহরের মানুষের মধ্যে কোভিড-১৯ এর টিকার আচরণগত নির্ধারকসমূহের গ্রহণযোগত্য নিরুপন।

সম্মতি পত্র

আমি..............., আমাকে প্রশ্নমালা ভিত্তিক গবেশনায় অংশগ্রহন করতে বলা হয়েছে। এটা আমি বুঝতে পেরেছি যে, এই প্রশ্নমালাটি তৈরি করা হয়েছে, "বাংলাদেশে ঢাকা শহরের মানুষের মধ্যে কোভিড-১৯ এর টিকার গ্রহনের আচরণগত নির্ধারকসমূহের গ্রহণযোগত্য নিরুপন" করার লক্ষ্যে। শিরনামে বিবৃত গবেষনার তথ্য সংগ্রহ করার জন্য।

এই গবেষণার উদ্দেশ্য ও সাধারণ তথ্যাবলি, কী ধরনরে প্রশ্ন করা হবে এবং প্রাপ্ত তথ্যগুলো কী কাজে আসবে, এগুলো আমাকে জানানো হয়েছে। আমি বুঝেছি যে, প্রশ্নগুলো একক ব্যক্তিকে জিজ্ঞেস করা হবে এবং এটা সম্পন্ন করতে ২০-২৫ মিনিট সময় লাগবে।

আমি স্বেচ্ছায় এই গবেষণায় অংশগ্রহন করেছি এবং অংশগ্রহন করা না করার ক্ষেত্রে আমি স্বাধীন এবং আমি চাইলে সাক্ষাৎকার চলাকালীন যে কোন সময় অংশগ্রহন বাতিল করতে পারবো। এবং এটাও আমি জানি, আমি চাইলে কোন সুনির্দিষ্ট প্রশ্নের উত্তর না দেওয়ার ক্ষেত্রে স্বাধীনতাও আমার আছে।

আমি এটা বুঝতে পেরেছি যে, আমার দেওয়া তথ্যগুলোর গোপনীয়তা বজায় রাখা হবে এবং শুধুমাত্র গবেষণার কাজে ব্যবহার করা হবে। এবং আমার নাম পরিচয় গোপন রাখা হবে। এই গবেষণায় অংশগ্রহণ করার কারণে আমি কোন ক্ষতির সম্মুখীন হবো না।

সাক্ষাৎকার থেকে প্রাপ্ত সকল তথ্যসমূহ, নোট, রেকর্ড নিরাপদ জায়গায় রাখা হবে। শুধুমাত্র গবেষক দলের সদস্যরা এগুলো গবেষণার কাজে ব্যবহারের সুযোগ পাবে।

আমি এটাও বুঝতে পেরেছি, এই গবেষণার ফলাফল আমার পরিচয় গোপন রেখে শুধুমাত্র গবেষণা কাজ, রিপোর্ট তৈরি এবং জার্নাল প্রকাশে ব্যবহার করা হবে।

উপরের তথ্যসমূহ আমি পড়েছি, নিচে আমার স্বাক্ষর প্রদান করে ফর্মটি জমাদানের মাধ্যমে এই গবেষণা কাজে অংশগ্রহণের জন্য সম্মতি প্রদান করলাম।

অংশগ্রহনকারীর নাম (দয়া করে আঙ্গুলের ছাপ দিন).............................

স্বাক্ষরঃ ....

তারিখঃ..........................

সাক্ষাৎকার গ্রহনকারীর নামঃ ....

সাক্ষরঃ ....

তারিখঃ ....

গ্রুপঃ ❑ ডুয়ার ❑ নন-ডুয়ার

ব্যারিয়ার এ্যানালাইসিস প্রশ্নাবলীঃ
বাংলাদেশের শহরে বসবাসকারী প্রাপ্তবয়স্কদের মধ্যে কোভিড-১৯ (করোনার) এর ভ্যাকসিনের গ্রহণযোগ্যতা

আচরন বিবৃতিঃ
শহরে বসবাসরত ১৮ বছর বা তদূর্ধ প্রাপ্তবয়স্ক মানুষ বলেছেন যে যখন কোভিড-১৯ (করোনার) এর ভ্যাকসিন/টীকা বিনামূল্যে পাওয়া যাবে, তখন তা গ্রহণ করতে তাদের ইচ্ছা রয়েছে।

ভূমিকা:

আসসালামওয়ালাইকুম / নমষ্কার, আমার নাম ... . ; কোভিড-১৯ (করোনার) এর ভ্যাকসিন/টীকা গ্রহন করা সংক্রান্ত মানুষের আগ্রহ নিয়ে যে দল গবেষণা করছে আমি সেই দলের একজন সদস্য। এখন পর্যন্ত অনেকগুলো ভ্যাকসিন/টীকা তৈরি করা হচ্ছে এবং সম্ভবত খুব দ্রুতই এগুলোর অনুমোদন হতে পারে। এই গবেষণায় এ সংক্রান্তা আলোচনা থাকবে এবং এই আলোচনাটি শেষ করতে প্রায় ২০ মিনিটের মতো সময় লাগবে। এই বিষয়ে আমি আপনার মতামত শুনতে চাই। এই গবেষণায় অংশগ্রহণ করা সর্ম্পূণ স্বেচ্ছামূলক; আপনি এতে অংশগ্রহণ করার সিদ্ধান্ত নিতে পারেন আবার নাও নিতে পারেন। আপনি যদি অংশগ্রহণ করতে না চান তাহলে কোন ধরনের সেবা প্রদান বন্ধ করা হবে না। যদি আপনি কোন উত্তর দিতে না চান তাহলে আপনি আমাকে বাদ দিতে বলবেন। আপনি যদি অংশগ্রহণ করার সিদ্ধান্ত নেন তাহলে আপনার নাম, পরিচয় এবং আপনি যা কিছু বলবেন সমস্ত কিছু গোপন রাখা হবে এবং কারো সাথে তা শেয়ার করা হবে না।

আপনি কি এই গবেষণায় অংশগ্রহণ করতে চান? (যদি তিনি সাক্ষাতকার দিতে রাজী হয় তাহলে নিচে তার স্বাক্ষর নিন। আর যদি না চান তাহলে উনাকে ধন্যবাদ দিন এবং অন্য আরেকজন সাক্ষাৎকার দাতা খুজুঁন।)

সেকশন এ - ডুয়ার/নন-ডুয়ার চিহ্নিতকরন প্রশ্নাবলী

১. আপনার বয়স কত ?
ক. ১৮ বছর অথবা এর বেশি (এখানে নির্দিষ্ট করুন কত বছর: ______)

খ. ১৮ বছরের কম → উত্তরদাতাকে ধন্যবাদ দিন, সাক্ষাৎকার শেষ করুন এবং পরবর্তী উত্তরদাতার খোঁজ করুন

গ. জানি না/উত্তর দিব না → উত্তরদাতাকে ধন্যবাদ দিন, সাক্ষাৎকার শেষ করুন এবং পরবর্তী উত্তরদাতার খোঁজ করুন

২. আপনি কি কখনও কোভিড-১৯ (করোনার) রোগটি সম্পর্কে শুনেছেন ?

ক. হ্যাঁ

খ. না → উত্তরদাতাকে ধন্যবাদ দিন, সাক্ষাৎকার শেষ করুন এবং পরবর্তী উত্তরদাতার খোঁজ করুন

গ. জানি না/ উত্তর দিব না → উত্তরদাতাকে ধন্যবাদ দিন, সাক্ষাৎকার শেষ করুন এবং পরবর্তী উত্তরদাতার খোঁজ করুন

৩. যদি আসন্ন মাসে কোভিড-১৯ (করোনার) এর ভ্যাকসিন/টীকা পাওয়া যায়, তবে ভ্যাকসিন/টীকাটি গ্রহণ করা আপনার জন্য কতটা সম্ভবপর হবে ? আপনি কি ভ্যাকসিন/টীকাটি গ্রহণ করবেন না কি গ্রহণ করবেন না ?

- *(যদি তারা বলেন "সম্ভবত")* পুনরায় প্রশ্ন করুন: আপনি কি বলবেন টীকাটি গ্রহণ করার অনেক সম্ভাবনা আছে না কি কিছুটা সম্ভাবনা আছে?
- *(যদি তারা বলেন "কোন সম্ভবনা নেই")* পুনরায় প্রশ্ন করুন: আপনি কি বলবেন টীকাটি গ্রহণ করার পক্ষে মোটেই সম্ভাবনা নেই নাকি কিছুটা সম্ভাবনা থাকলেও থাকতে পারে?

ক. অনেক সম্ভাবনা / অবশ্যই টীকাটি গ্রহণ করবে
খ. কিছুটা সম্ভাবনা আছে
গ. কিছুটা অসম্ভাবনা থাকতে পারে
ঘ. কোন সম্ভাবনা নেই / টীকাটি গ্রহণ করবো না
ঙ. জানি না
চ. উত্তর দিব না → উত্তরদাতাকে ধন্যবাদ দিন এবং সাক্ষাৎকার শেষ করুন
[যদি তারা বলেন "অবশ্যই", তবে অনেক সম্ভাবনায় টিক দিন। যদি তারা বলেন অবশ্যই না, তবে মোটেই সম্ভাবনা নেই - তে টিক দিন]

ডুয়ার/নন-ডুয়ার শ্রেণীবিন্যাস টেবিল

**ডুয়ার**<br>(নীচের সবগুলো প্রযোজ্য)	**নন-ডুয়ার**<br>(নীচের যেকোন একটি প্রযোজ্য)	**সাক্ষাৎকার নিবেন না**<br>(নীচের যেকোন একটি প্রযোজ্য)
প্রশ্ন ১ = ক	প্রশ্ন ১ = ক	প্রশ্ন ১ = খ অথবা গ
প্রশ্ন ২ = ক	প্রশ্ন ২ = ক	প্রশ্ন ২ = খ অথবা গ
প্রশ্ন ৩ = ক অথবা খ	প্রশ্ন ৩ = গ, ঘ অথবা ঙ	প্রশ্ন ৩ = চ

গ্রুপঃ ❑ ডুয়ার ❑ নন-ডুয়ার

আচরন ব্যাখ্যা

নিম্নলিখিত প্রশ্নগুলেতে, আমি আপনাকে একটি ভ্যাকসিন/টীকা দেওয়ার বিষয়ে জিজ্ঞাসা করব যা কোভিড-১৯ (করোনার) রোগ প্রতিরোধে সহায়তা করছে। যখন আমি এটা বলছি, তখন আমি আপনার বিষয়ে স্বাস্থ্যকর্মীর কাছ থেকে বিনামূল্যে একটি কোভিড-১৯ (করোনার) ভ্যাকসিন/টীকা আনার বা গ্রহণ করার কথা বলছি যখন সরকার বা স্বাস্থ্য কর্মকর্তাগন আপনার এলাকায় অনুমোদিত কোভিড-১৯ (করোনার) ভ্যাকসিন/টীকা পাওয়া যাচ্ছে বলে ঘোষনা করেছেন।

প্রাথমিক তথ্যাবলীঃ

সাক্ষাৎকার গ্রহণকারীর নামঃ ____________________________ প্রশ্নপত্র নংঃ ______ তারিখঃ ____/____/____

এলাকাঃ ____________________________ উত্তরদাতার লিঙ্গঃ _______________________________

শিক্ষাগত যোগ্যতাঃ _________________পেশাঃ _________________মোবাইল ফোন নংঃ _______________________

সেকশন বি - গবেষনামূলক প্রশ্নাবলী

(অনুমিত স্ব-ক্ষমতা বা দক্ষতা/Perceived Self-efficacy)

১. যদি আগামী কয়েক মাসের মধ্যে কোভিড-১৯ (করোনার) এর কোনও ভ্যাকসিন/টীকা বিনামূল্যে পাওয়া যায়, তাহলে কোন কোন বিষয়গুলোর কারনে তা গ্রহন করতে আপনার জন্য সহজ হবে ?
(সকল উত্তর নীচে লিখুন এবং প্রোব করুন "আর কি কি")

২. যদি আগামী কয়েক মাসের মধ্যে কোভিড-১৯ (করোনার) এর কোনও ভ্যাকসিন/টীকা বিনামূল্যে পাওয়া যায়, তাহলে কোন কোন বিষয়গুলোর কারনে তা গ্রহন করতে আপনার জন্য কঠিন হবে ?
(সকল উত্তর নীচে লিখুন এবং প্রোব করুন "আর কি কি")

(উপলব্ধ ইতিবাচক ফলাফল/ Perceived Positive Consequences)

৩. যদি আগামী কয়েক মাসের মধ্যে কোভিড-১৯ (করোনার) এর কোনও ভ্যাকসিন/টীকা বিনামূল্যে পাওয়া যায়, তাহলে তা গ্রহন করার ফলে আপনার কি কি উপকার হবে?
(সকল উত্তর নীচে লিখুন এবং প্রোব করুন "আর কি কি")

(উপলব্ধ নেতিবাচক ফলাফল/ Perceived Negative Consequences)

৪. যদি আগামী কয়েক মাসের মধ্যে কোভিড-১৯ (করোনার) এর কোনও ভ্যাকসিন/টীকা বিনামূল্যে পাওয়া যায়, তাহলে তা গ্রহন করার ফলে আপনার কি কি অসুবিধা/সমস্যা হতে পারে ?
(সকল উত্তর নীচে লিখুন এবং প্রোব করুন "আর কি কি")

(উপলব্ধ সামাজিক রীতি-নীতি/ Perceived Social Norms)

৫. যদি আগামী কয়েক মাসের মধ্যে কোভিড-১৯ (করোনার) এর কোনও ভ্যাকসিন/টীকা আপনার এলাকায় বিনামূল্যে পাওয়া যায়, তাহলে আপনার জানা মতে আপনার এলাকার কি পরিমাণ মানুষ এই ভ্যাকসিন/টীকাটি গ্রহণ করবে ?

ক. বেশীরভাগ জনগন এটি গ্রহণ করবে

খ. অর্ধেক জনগন এটি গ্রহণ করবে

গ. খুবই সামান্য পরিমান এটি গ্রহণ করবে

ঘ. জানি না/বলতে চায়নি

[যদি তারা বলে "সবাই" বেশীরভাগ -এ টিক দিন, যদি বলে "কেউ না" তাহলে খুবই সামান্য তে টিক দিন]

৬. আপনি কি মনে করেন যে, যদি আগামী কয়েক মাসের মধ্যে কোভিড-১৯ (করোনার) এর কোনও ভ্যাকসিন/টীকা বিনামূল্যে পাওয়া যায় তাহলে আপনার পরিবারের সদস্য এবং বন্ধুবান্ধব কি আপনাকে এই টীকাটি গ্রহণ করতে উবুদ্ধ করবে?

ক. হ্যাঁ

খ. না

গ. জানি না/উত্তর দিব না

৭. আপনি কি মনে করেন যে, যদি আগামী কয়েক মাসের মধ্যে কোভিড-১৯ (করোনার) এর কোনও ভ্যাকসিন বিনামূল্যে পাওয়া যায়, আপনার সমাজের নেতা এবং ধর্মীয় নেতৃবৃন্দ আপনাকে টীকাটি গ্রহণ করতে দিতে রাজি হবে?

ক. হ্যাঁ

খ. না

গ. জানি না/ উত্তর দিব না

৮. যদি আগামী কয়েক মাসের মধ্যে কোভিড-১৯ (করোনার) এর কোনও ভ্যাকসিন বিনামূল্যে পাওয়া যায়, তাহলে তা গ্রহন করার জন্য আপনাকে **কে কে সমর্থন করবেন (কারা আপনাকে অনুমতি দিবে)** ?

(সকল উত্তর নীচে লিখুন এবং প্রোব করুন "আর কে ?")

৯. যদি আসন্ন মাসে কোভিড-১৯ (করোনার) এর কোনও ভ্যাকসিন বিনামূল্যে পাওয়া যায়, তাহলে তা গ্রহন করার জন্য আপনাকে **কে কে সমর্থন করবে না (কারা আপনাকে অনুমতি দিবে না)?**

(সকল উত্তর নীচে লিখুন এবং প্রোব করুন "আর কে ?")

১০. যদি কোন ডাক্তার অথবা নার্স আপনাকে কোভিড-১৯ (করোনার) এর ভ্যাকসিন/টীকা গ্রহন করার জন্য সুপারিশ করেন, তবে আপনার সেটি গ্রহণ করার সম্ভাবনা কতটুকু?

ক. অনেক সম্ভাবনা
খ. মোটামুটি সম্ভাবনা
গ. সম্ভাবনা নেই
ঘ. জানি না/উত্তর দিব না

(উপলব্ধ সুযোগ-সুবিধা/ *Perceived Access)*

১১. যদি আগামী কয়েক মাসের মধ্যে কোভিড-১৯ (করোনার) এর কোনও ভ্যাকসিন বিনামূল্যে পাওয়া যায়, তাহলে আপনি কি মনে করেন যে তা আপনার বাড়ি থেকে ৩০ মিনিটের হাঁটা পথের দুরত্বের মধ্যে তা পাওয়া যাবে?

ক. হ্যাঁ
খ. না
গ. জানি না/উত্তর দিব না

১২. যদি আগামী কয়েক মাসের মধ্যে কোভিড-১৯ (করোনার) এর কোনও ভ্যাকসিন বিনামূল্যে পাওয়া যায়, তাহলে আপনার মতে, ভ্যাকসিন/টীকাটি নিতে মানুষকে গড়ে কত ঘন্টা বা কত মিনিট লাইনে দাঁড়িয়ে অপেক্ষা করতে হতে পারে?

(উত্তরগুলো ঘন্টা ও মিনিটে লিখুন)

____ ঘন্টা ____ মিনিট

(উপলব্ধ সংবেদনশীলতা/ঝুঁকি/ *Perceived Susceptibility / Perceived Risk)*

১৩. আপনার জানামতে, আপনার এলাকায় শতকরা কতজন মানুষ কোভিড-১৯ (করোনায়) রোগে আক্রান্ত হয়েছে?

ক. অনেক মানুষ আক্রান্ত
খ. কিছু মানুষ আক্রান্ত
গ. খুব সামান্য মানুষ আক্রান্ত
ঘ. কেউ আক্রান্ত হয়নি
ঙ. জানি না/উত্তর দিব না

১৪. আপনার মতে, আগামী তিন মাসের মধ্যে আপনি অথবা আপনার পরিবারের সদস্যদের কেউ কোভিড-১৯ (করোনায়) রোগে আক্রান্ত হওয়ার সম্ভাবনা কতটুকু?

ক. খুবই সম্ভাবনা আছে
খ. মোটামুটি সম্ভাবনা আছে
গ. মোটেই সম্ভাবনা নেই
ঘ. জানি না/ উত্তর দিব না

১৫. কোভিড-১৯ (করোনায়) রোগে আক্রান্ত হওয়ার ব্যাপারে আপনি কতটা উদ্বিগ্ন

ক. মোটেই উদ্বিগ্ন নন
খ. সামান্য উদ্বিগ্ন
গ. মোটামুটি উদ্বিগ্ন
ঘ. খুবই উদ্বিগ্ন

(উপলব্ধ তীব্রতা/ *Perceived Severity)*

১৬. আপনি অথবা আপনার পরিবারের কোন সদস্য যদি কোভিড-১৯ (করোনায়) রোগে আক্রান্ত হন, তবে তা কতটা গুরুতর হতে পারে?

ক. খুবই গুরুতর হবে
খ. মোটামুটি গুরুতর হবে
গ. মোটেই গুরুতর হবে না
ঘ. জানি না/ উত্তর দিব না

(কার্যক্রমের উপলব্ধ ফলপ্রসূতা/ Perceived Action Efficacy)

১৭. আপনি যদি কোভিড-১৯ (করোনার) ভ্যাকসিন/টীকা গ্রহণ করেন তার পরে আপনার কোভিড-১৯ (করোনায়) রোগে আক্রান্ত হওয়ার সম্ভাবনা কতটুকু?

ক. খুবই সম্ভাবনা আছে
খ. মোটামুটি সম্ভাবনা আছে
গ. মোটেই সম্ভাবনা নেই
ঘ. জানি না/ উত্তর দিব না

১৮. আগামী কয়েক মাসের মধ্যে যদি কোভিড-১৯ (করোনার) এর একটি নতুন ভ্যাকসিন/টীকা বিনামূল্যে পাওয়া যায়, তবে সেটিতে আপনি কতটা বিশ্বাস করবেন?

ক. মোটেও বিশ্বাস করবো না
খ. কিছুটা বিশ্বাস করবো
গ. মোটামুটি বিশ্বাস করবো
ঘ. অনেক বিশ্বাস করবো
ঙ. জানি না/ উত্তর দিব না

১৯. "কিছু লোক ভ্যাকসিন বা টীকার নিরাপত্তা সম্পর্কে উদ্বিগ্ন - যেমন, অনেকেই মনে করেন এই টীকা গ্রহন করার ফলে গুরুতর পার্শ্ব-প্রতিক্রিয়া হওয়ার সম্ভাবনা- আবার, কিছু লোক উদ্বিগ্ন নন। একটি কোভিড-১৯ (করোনার) ভ্যাকসিন/টীকা গ্রহণ করা আপনার জন্য কতটা **নিরাপদ** বলে আপনি মনে করেন?

ক. মোটেও নিরাপদ নয়
খ. মোটামুটি নিরাপদ
গ. খুবই নিরাপদ
ঘ. জানি না/ উত্তর দিব না

২০. "কেউ যদি কোভিড-১৯ এ আক্রান্ত হয় তাহলে তার আর করোনা ভাইরাসের টীকা বা ভ্যাকসিন নেওয়ার দরকার নেই।" আপনি এই মতামতের সাথে আপনি কতটুকু একমত? (যদি তারা এর সাথে একমত হয় তাহলে জিজ্ঞেস করুন কতটা একমত? বেশি, কম না-কি অনেক বেশি)।

ক. কম একমত।
খ. অনেক বেশি একমত।
গ. কিছুটা দ্বিমত।
ঘ. একেবারেই একমত না।
ঙ. জানি না/বলবো না।

২১. "বেশিরভাগ মানুষ কখনো না কখনো করোনা ভাইরাস বা কোভিড-১৯ এ আক্রান্ত হবেই, এতে করে সবার এন্টিবডি তৈরি হয়ে যাবে। সুতরাং করোনা ভাইরাসের টীকা/ভ্যাকসিন নেওয়ার দরকার নেই।"

আপনি এই মতামতের সাথে কতটুকু একমত? (যদি তারা এর সাথে একমত হয় তাহলে জিজ্ঞেস করুন কতটা একমত? বেশি, কম না-কি অনেক বেশি)।

ক. খুব বেশি একমত না।
খ. অনেক বেশি একমত।
গ. কিছুটা দ্বিমত।
ঘ. একেবারেই একমত না।
ঙ. জানি না/বলবো না।

(উপলব্ধ ঐশ্বরিক ইচ্ছা/Perceived Divine Will)

২২. আপনি কি মনে করেন আল্লাহ্ বা ঈশ্বর (দেব-দেবতা) মানুষের জন্য কোভিড-১৯ (করোনার) ভ্যাকসিন/টীকা গ্রহণ করা অনুমোদন অথবা অস্মীকার করেন?

ক. আমি বিশ্বাস করি ঈশ্বর (আল্লাহ্ বা দেবতাগন) অনুমোদন করেন
খ. আমি বিশ্বাস করি ঈশ্বর (আল্লাহ্ বা দেবতাগন) অনুমোদন করেন না
গ. আমি বিশ্বাস করি ঈশ্বর (আল্লাহ্ বা দেবতাগন) অনুমোদন অথবা অস্মীকার করেন না
ঘ. জানি না/ উত্তর দিব না

২৩. আপনি কি নিম্লোক্ত বিবৃতির সাথে সম্মত বা সম্মত নন - "আমি কোভিড-১৯ (করোনায়) রোগে আক্রান্ত হই বা না হই, তা সম্পুর্নভাবে ঈশ্বরের (আল্লাহ্ বা দেবতাগনের) ইচ্ছা বা পরিকল্পনা। আমি যদি কোন পদক্ষেপ গ্রহন করিও তাতে কোভিড-১৯ (করোনায়) এ আক্রান্ত হওয়ার ক্ষেত্রে সামান্যই প্রভাব ফেলবে।"

(যদি "সম্মত" হয়)ঃ আপনি কি সামান্য সম্মত না কি অনেকটাই সম্মত?
ক. সামান্য সম্মত
খ. অনেকটাই সম্মত

(যদি "অসম্মত" হয়)ঃ আপনি কি সামান্য অসম্মত না কি অনেকটাই অসম্মত?
ক. সামান্য অসম্মত
খ. অনেকটাই অসম্মত

(সংস্কৃতি/Culture)

২৪. যদি আগামী কয়েক মাসের মধ্যে কোভিড-১৯ (করোনার) এর কোনও ভ্যাকসিন/টীকা বিনামূল্যে পাওয়া যায়, তবে এমন কোনও সাংস্কৃতিক বা ধর্মীয় কারন রয়েছে কি যার জন্য আপনি ভ্যাকসিন/টীকাটি গ্রহণ করবেন না?

ক. হ্যাঁ
খ. না → প্রশ্ন নং ২৪ এ যান
গ. জানি না/বলতে চায়নি → প্রশ্ন নং ২৪ এ যান

২৫. সেই কারনগুলো কি কি?
(সবগুলো উত্তর লিখুন। প্রশ্ন করুন "আর কি কি?")

(অন্যান্য সম্ভাব্য সম্পর্ক)

২৬. যদি এমন কোন প্রশ্ন থাকে যার উত্তর আপনি দিতে চান না, তাহলে আপনি আমাকে সেটা এড়িয়ে যেতে বলতে পারেন। সরকারী কর্মকর্তা/কর্মচারী এবং রাজনীতিবিদগন কোভিড-১৯ (করোনার) ভ্যাকসিন/টীকার নিরাপত্তা ও কার্যকারীতা সম্পর্কে যেসব তথ্য দেন তাতে কি আপনি বিশ্বাস করেন?

(যদি "হ্যাঁ" হয়)ঃ আপনি কি বলছেন যে, সরকারী কর্মকর্তা/কর্মচারী এবং রাজনীতিবিদগন যেসব তথ্য দেন তা কি আপনি মোটামুটি বিশ্বাস করবেন, না কি অনেক বেশি বিশ্বাস করেন?

(যদি "না" হয়)ঃ আপনি কি বলছেন যে, সরকারী কর্মকর্তা/কর্মচারী এবং রাজনীতিবিদগন যেসব তথ্য দেন তাতে কি আপনি মোটামুটি কম পর্যায়ের বিশ্বাস করবেন, না কি অনেক কম পর্যায়ের বিশ্বাস করবেন?

ক. অনেক কম পর্যায়ের বিশ্বাস করি
খ. মোটামুটি কম পর্যায়ের বিশ্বাস করি
গ. মোটামুটি উচ্চ পর্যায়ের বিশ্বাস করি
ঘ. অনেক বেশি বিশ্বাস করি
ঙ. জানি না/ উত্তর দিব না

২৭. ধর্মীয় নেতৃবৃন্দ কোভিড-১৯ (করোনার) ভ্যাকসিন/টীকার নিরাপত্তা ও কার্যকারীতা সম্পর্কে যেসব তথ্য দেন তাতে কি আপনি বিশ্বাস করবেন?

(যদি "হ্যাঁ" হয়)ঃ আপনি কি বলছেন যে, ধর্মীয় নেতৃবৃন্দ যেসব তথ্য দেন তাতে কি আপনি মোটামুটি উচ্চ পর্যায়ের বিশ্বাস করবেন, না কি অনেক উচ্চ পর্যায়ের বিশ্বাস করবেন?

(যদি "না" হয়)ঃ আপনি কি বলছেন যে, ধর্মীয় নেতৃবৃন্দ যেসব তথ্য দেন তাতে কি আপনি মোটামুটি কম পর্যায়ের বিশ্বাস করবেন, না কি অনেক কম পর্যায়ের বিশ্বাস করবেন?

ক. অনেক কম পর্যায়ের বিশ্বাস করি
খ. মোটামুটি কম পর্যায়ের বিশ্বাস করি
গ. মোটামুটি উচ্চ পর্যায়ের বিশ্বাস করি
ঘ. অনেক বেশি বিশ্বাস করি
ঙ. জানি না/উত্তর দিব না

২৮. আপনি কি এমন কিছু দেখেছেন বা শুনেছেন যা আপনাকে বা অন্যদের কোভিড-১৯ (করোনার) ভ্যাকসিন/টীকা (যা দ্রুতই পাওয়া যাবে) পাওয়ার জন্য বাধা দেয়?
ক. হ্যাঁ
খ. না → সাক্ষাৎকার শেষ করুন এবং উত্তরদাতাকে ধন্যবাদ দিন
গ. জানি না/উত্তর দিব না → সাক্ষাৎকার শেষ করুন এবং উত্তরদাতাকে ধন্যবাদ দিন

২৯. এমন কোন কোন বিষয় সম্পর্কে আপনি কি শুনেছেন যা আপনাকে বা অন্যদেরকে কোভিড-১৯ (করোনার) ভ্যাকসিন/টীকা পেতে/নিতে বাধা দেবে?
[নীচে তারা যা শুনেছে তার তার তালিকা তৈরী করুন এবং প্রশ্ন করুন "আর কি?"]

উত্তরদাতাকে তার সময়ের জন্য ধন্যবাদ দিন
