# Supplementary material for: Exploring the behavioral determinants of COVID-19 vaccine acceptance among an urban population in Bangladesh: Implications for behavior change interventions
Source: PLoS One. 2021 Aug 23;16(8):e0256496. doi: 10.1371/journal.pone.0256496 (PMC8382171; doi:10.1371/journal.pone.0256496)
Supplement: S4 File — (PDF) [file pone.0256496.s004.pdf]

Understanding behavioral determinants of COVID-19 vaccine acceptance among urban population in Dhaka, Bangladesh.

Consent form

I ……………, understand that I am being asked to participate in a questionnaire-based study. It is my understanding that this questionnaire has been designed to gather information about the study titled as stated "Understanding behavioral determinants of COVID-19 vaccine acceptance among urban population in Dhaka, Bangladesh".

I have been given general information about the study that includes the objectives of the study, type of questions to be asked and the use of information. I understand that the questions will be asked in person and will take approximately 20-25 minutes to complete.

I understand that my participation in this study is completely voluntary and I am free to decide whether I participate in the study or not and I can withdraw my participation at any point of the interview. I also understand that I have full freedom to not answer any specific question if I do not want to answer.

I understand that any information I provide will be kept confidential and used only for research purpose and will not be used any way that can identify me. I have been also explained that no harm will be occurred as a result of participation in the study.

I understand that all responses, notes and records will be kept in a secured location by using a sealed locker. Only the research team members will have the access on those for analysis purpose.

I also understand that the results of this study will be used exclusively for the purpose of the study, report preparation and journal publication without jeopardizing my identification.

I have read the information above. By signing below and returning this form, I am consenting to participate in this study.

Respondent name (Please print): …………………….

Signature: ………………….

Date: …………………………

Interviewer name: …………………………

Signature: ……………….

Date: ………………………

Group: ❑ Doer ❑ Non-Doer

Barrier Analysis Questionnaire:

Behavior Statement

Adult men and women 18 years or older of age living in Dhaka urban areas have stated intention to get a COVID-19 vaccine when one is available to them free of charge.

Section A - Doer/Non-doer Screening Questions

1. What is your age?
   ❑ a. 18 years or older (specify age in years here: ____)
   ❑ b. Less than 18 years → *Thanks the respondent, end the interview and look for another respondent*
   ❑ c. Don't Know / Won't say → *Thank the respondent, end the interview and look for another respondent*

2. Have you ever heard of the disease COVID-19 or Coronavirus?
   ❑ a. Yes
   ❑ b. No → *Thank the respondent, end the interview and look for another respondent*

3. If a COVID-19 vaccine was available to you in the coming month, how likely would you be to get the vaccine, that is to go for vaccination? Would you say you are likely or unlikely to get the vaccine?
   - ***(If they say "Likely:")*** *probe further and ask:* Would you say you are Very Likely or Somewhat Likely to get the vaccine?
   - ***(If they say "Unlikely:")*** *probe further and ask:* Would you say you are Very unlikely or Somewhat Unlikely to get the Vaccine?

   ❑ a. Very Likely / Definitely would get the vaccine
   ❑ b. Somewhat likely
   ❑ c. Somewhat unlikely
   ❑ d. Very unlikely / Definitely would not get the vaccine
   ❑ e. Don't Know
   ❑ f. Won't say → *Thank the person and end the interview*

[If they say "definitely", mark Very Likely. If they say, "definitely not," mark Very Unlikely.]

DOER /NON-DOER CLASSIFICATION TABLE

**DOER** (**ALL** of the following)	**Non-Doer** (**any ONE** of the following)	**Do Not Interview** (**any ONE** of the following)
Question 1 = A		Question 1 = B or C
Question 2 = A		Question 2 = B
Question 3 = A or B	Question 3 = C, D or E	Question 3 = F

Group: ❑ Doer ❑ Non-doer

Behavior Explanation
In the following questions, I am going to be asking you about getting a vaccine that helps prevent the COVID-19 disease. When I say this, I am talking about you going to get a COVID-19 vaccine, free of charge, from a medical worker when the government or health officials announce that an approved COVID-19 vaccine is available in your area.

Demographic Data

Interviewer's Name: ___________________ Questionnaire No.: ____Date: ___/___/___

Area/Community: ______________ Mobile Phone: _____________________ Gender: ______

Age (in years): Level of education:

Section B – Research Questions

(Perceived Self-efficacy)

1. If a vaccine for COVID -19 were available to you in the coming month free of charge, what might make it ***easier*** for you to get that vaccine?

(Write all responses below. Probe with "What else?")

2. If a vaccine for COVID -19 were available to you in the coming month free of charge, what might make it ***difficult*** for you to get that vaccine?

(Write all responses below. Probe with "What else?")

(Perceived Positive Consequences)

3. If a vaccine for COVID -19 were available to you in the coming month free of charge, what would be the ***advantages*** of getting that vaccine?

(Write all responses below. Probe with "What else?")

(Perceived Negative Consequences)

4. If a vaccine for COVID -19 were available to you in the coming month free of charge, what would be the ***disadvantages*** of getting that vaccine?

(Write all responses below. Probe with "What else?")

(Perceived Social Norms)

5. If a vaccine for COVID -19 were available to **people in your community** in the coming month free of charge, **what portion of the people you know do you think would get the vaccine**? Would you say that most people would get it, about half of people would get it, or very few people would get it?

- ❑ a. Most people would get the vaccine
- ❑ b. About half of people would get the vaccine
- ❑ c. Very few people would get the vaccine
- ❑ d. Don't Know / Won't say

[If they say everyone, choose "most." If they say "no one," mark "few people."]

6. If a vaccine for COVID -19 were available to you in the coming month free of charge, who are the people that ***would approve*** of your getting the vaccine?

(Write all responses below. Probe with "Who else?")

7. If a vaccine for COVID -19 were available to you in the coming month free of charge, who are the people that **would disapprove** of your getting the vaccine?

(Write all responses below. Probe with "Who else?")

8. Do you think that **most of your close family and friends would want you to get a COVID-19 vaccine** if it was available to you in the coming month free of charge?

- ❑ a. Yes
- ❑ b. No
- ❑ c. Don't Know / Won't say

9. Do you think that **most of your community leaders and religious leaders would want you to get a COVID-19 vaccine** if it was available to you in the coming month free of charge?

❑ a. Yes
❑ b. No
❑ c. Don't Know / Won't say

10. If a **doctor or nurse recommended** that you get the COVID-19 vaccine, how likely would you be to get it? Would you say very likely, somewhat likely, or not likely to get it?
❑ a. Very likely
❑ b. Somewhat likely
❑ c. Not likely
❑ d. Don't Know / Won't say

(Access)

11. How difficult is it for you **to get the clinic** where vaccines are normally offered? Would you say it's very difficult, somewhat difficult or not difficult at all?
❑ a. Very difficult
❑ b. Somewhat difficult
❑ c. Not difficult at all

(Perceived Susceptibility / Perceived Risk)

11. To your knowledge, what proportion of people in the community where you live have had COVID-19 disease? Would you say that **very many people, some people, very few people, or no one has had COVID-19 where you live?**
❑ a. Very many people
❑ b. Some people
❑ c. Very few people
❑ c. No one
❑ d. Don't Know / Won't say

14. How **likely** do you think it is that someone who lives in your household will contract COVID-19 over the next three months? Would you say it's very likely, somewhat likely or not likely at all?
❑ a. Very likely
❑ b. Somewhat likely
❑ c. Not likely at all
❑ d. Don't Know / Won't say

15. How **concerned** are you about getting COVID-19? Would you say that you are not at all concerned, a little concerned, moderately concerned, or very concerned?
❑ a. Not at all concerned
❑ b. A little concerned
❑ c. Moderately concerned
❑ d. Very concerned

(Perceived Severity)

16. How **serious** would it be if someone who lives in your household contracted COVID-19? Would you say it would be very serious, somewhat serious, or not serious at all?

❑ a. Very serious
❑ b. Somewhat serious
❑ c. Not serious at all
❑ d. Don't Know / Won't say

(Perceived Action Efficacy)

17. If you were to get the vaccine for COVID-19, how likely would it be that you would **get COVID-19 Disease** after that? Very likely, somewhat likely, or not likely at all?

❑ a. Very likely
❑ b. Somewhat likely
❑ c. Not likely at all
❑ d. Don't Know / Won't say

18. How much would you **trust** a new COVID-19 vaccine if it were were available to you in the coming month free of charge? Would you say you would not trust it at all, trust it a little, trust it a moderate amount, or trust it a lot?

❑ a. Not trust it at all
❑ b. Trust it a little
❑ c. Trust it a moderate amount
❑ d. Trust it a lot
❑ e. Don't Know / Won't say

19. Some people are concerned about the safety of vaccines – such as the likelihood for having a serious reaction – and some people are not. How **safe** do you think it would be for you to get a COVID-19 vaccine? Would you say it would not be safe at all, it would be mostly safe, or it would be very safe for you to get the vaccine?

❑ a. Not safe at all
❑ b. Mostly safe
❑ c. Very safe
❑ d. Don't Know / Won't say

20. If one has been infected with COVID-19, vaccination with the COVID-19 vaccine is unnecessary." Would you say you agree or disagree with that statement?
• (If they say "Agree:") probe further and ask: Would you say you Agree a little or Agree a lot?
• (If they say "Disagree:") probe further and ask: Would you say you Disagree a little or Disagree a lot?

❑ a. Agree a little
❑ b. Agree a lot

❑ c. Disagree a little
❑ d.Disagree a lot
❑ e. Don't Know / Won't say

21. Consider the following statement: Most people will eventually get infected with COVID-19, so getting the COVID-19 vaccine is unnecessary." Would you say you agree or disagree with that statement?

• (If they say "Agree:") probe further and ask: Would you say you Agree a little or Agree a lot?
• (If they say "Disagree:") probe further and ask: Would you say you Disagree a little or Disagree a lot?

❑ a. Agree a little
❑ b. Agree a lot
❑ c. Disagree a little
❑ d.Disagree a lot
❑ e. Don't Know / Won't say

(Perceived Divine Will)

22. Do you think that **God (or Allah or the gods) approve(s) or disapprove(s) of** people getting a COVID-19 vaccine?
❑ a. I believe that God approves
❑ b. I believe that God does not approve
❑ c. I believe that God does not approve or disapprove
❑ d. Don't Know / Won't say

23. Do you agree or disagree with the following statement? "Whether I get COVID-19 or not is purely **a matter of God's will or chance**. The actions I take will have little bearing on whether or not I get COVID-19."
(If "Agree"): "Do you agree a little or agree a lot?"
❑ a. Agree a little
❑ b. Agree a lot
(If "Disagree"): "Do you disagree a little or disagree a lot?"
❑ c. Disagree a little
❑ d. Disagree a lot

(Culture)

24. If a vaccine for COVID -19 were available to you in the coming month free of charge, are there any **cultural or religious reasons** that you would **not** get the vaccine?

❑ a. Yes
❑ b. No → ***Go to question #24***
❑ c. Don't Know / Won't say → ***Go to question #24***

25. What are those reasons?

(Write all responses below. Probe with "Who else?")

(Other possible correlates)

26. What level of education did you complete?

❑ a. Some primary, but did not complete primary
❑ b. Completed primary
❑ c. Some secondary, but did not complete secondary
❑ d. Completed secondary
❑ e. Some college, but did not complete college
❑ f. Completed college
❑ g. Some graduate work or completed graduate degree
❑ h. Other
❑ i. Don't know / Won't say

27. Remember, if there is any question that you do not want to answer, you can just ask me to skip the question. Would you **trust the information** that government representatives and politicians provide on the safety and effectiveness of COVID-19 vaccines?

- ***(If "Yes", ask:)*** Would you say you have a **somewhat high level of trust or a very high level of trust** in the information that government workers or politicians provide on COVID-19 vaccines?
- ***(If "No", ask:)*** Would you say you have a **somewhat low level of trust, or a very low level of trust** in the information that government workers or politicians provide on COVID-19 vaccines?

❑ a. Very low level of trust
❑ b. Somewhat low level of trust
❑ c. Somewhat high level of trust
❑ d. Very high level of trust
❑ e. Don't know / Won't say

28. Would you **trust the information** that religious leaders provide on the safety and effectiveness of COVID-19 vaccines?

- ***(If "Yes", ask:)*** Would you say you have a **somewhat high level of trust or a very high level of trust** in the information that religious leaders provide on COVID-19 vaccines?
- ***(If "No", ask:)*** Would you say you have a **somewhat low level of trust, or a very low level of trust** in that information that religious leaders provide on COVID-19 vaccines?

❑ a. Very low level of trust
❑ b. Somewhat low level of trust
❑ c. Somewhat high level of trust
❑ d. Very high level of trust

❑ e. Don't know / Won't say

29. Have you seen or heard of anything that would stop you or others from seeking to get the COVID-19 Vaccine (which is expected soon)?

❑ a. Yes
❑ b. No → ***End the interview and thank the respondent.***
❑ c. Don't Know / Won't say → ***End the interview and thank the respondent.***

30. What have you heard that would stop you or others from seeking to get the COVID-19 vaccine?
(List all the things below that they have heard. Say "what else?" after each response.)

THANK THE RESPONDENT FOR HIS /HER TIME!
