# Supplementary material for: Exploring the behavioral determinants of COVID-19 vaccine acceptance among an urban population in Bangladesh: Implications for behavior change interventions
Source: PLoS One. 2021 Aug 23;16(8):e0256496. doi: 10.1371/journal.pone.0256496 (PMC8382171; doi:10.1371/journal.pone.0256496)
Supplement: Attachment — Submitted filename: Comments of reviewer I.docx [file pone.0256496.s005.docx]

**Comments of reviewer 1**

Thank you for referring this **manuscript** for revision. The idea is interesting. The article studies an urgent problem, investigating the behavioral determinants of COVID-19 vaccine acceptance among an Urban Population in Dahka, the Capital of Bangladesh. Authors believe that identifying these determinants will help in designing a suitable strategy to increase the acceptance and uptake rate of vaccination in Bangladesh. Individuals have several reasons for adopting or resisting certain behavior. It is important to identify these barriers or facilitators which influence somebody’s willingness to get health services.

**Introduction** of this manuscript demonstrating the size of the problem of hesitancy or refusing COVID-19 vaccination in different countries. However, on displaying the situation in Bangladesh authors reported that 81% of urban people would get vaccinated when a COVID-19 vaccine is available (9). So, why did authors choose to investigate the Urban population despite this high rate of acceptance among them?

I think authors must search for another reference showing the size of the problem in Bangladesh.

If population in rural areas was investigated, it might be more sounding.

At the end of introduction, the authors stated that their study included different societal structures (Line 107, page 4) which was not presented in data.

**Subjects and methods:** The paragraph from line (112-117, page 6) needs revision and rephrasing. The duration of the study is very short (one week).

**Study tool:** This section describing Barrier analysis is copied from a previous article. Applying a plagiarism checker is essential.

**Questionnaire development:** Authors stated that this study modified the standardized Barrier Analysis questionnaire. They did not mention these modifications. They omitted one determinant only from the 12 ones without explanation. These items could be summarized to the four essential determinants.

The paragraph from line (0- 12, page 9) is also copied from a previous literature (Determinants of COVID-19 Vaccine Acceptance in Six Lower- and Middle-Income Countries). Applying a plagiarism checker is essential.

**Sample:** sample size is small. Building an intervention plan requires a representative sample from different societal positions.

Authors did not demonstrate the age range nor the sex ratio of the recruited subjects.

Authors did not declare the type of random sampling technique.

It was better to summarize the questionnaire, increase the sample size and include some rural districts.

**Results:** This section is confusing. A lot of tables, with a lot of data, with several titles and subtitles. I think the lengthy questionnaire with many open-ended questions yielded in numerous data.

Characteristics of the studied participants must be presented in a separate table showing age categories, sex ratio, educational levels, occupation, and social class which are important variables affecting subject’s behavior.

Why did items related to Perceived Social Norms is divided in table 1 and table 4?

Could the number of tables be less or replaced by figures for some determinants?

I could not identify which beliefs were most highly associated with acceptance and non-acceptance with COVID-19 vaccine.

Could authors do a regression analysis to conclude the predictors of each behavior, whether acceptance or non-acceptance?

**Discussion:** is well written, organized, updated, and provided a good model to increase the rate of COVID-19 vaccine acceptance among Bangladesh population. This strategy could be applied in other developing countries.

**Conclusion:** is written in a general way and not concentrating on the main findings

**References:** authors may need to revise this section to correct incomplete references and delete repeated ones as number 15 and 19.

After careful consideration, I think that this manuscript will likely be suitable for publication if it is revised to address the points mentioned before (Increasing the sample size, including rural plus urban population, summarizing the open-ended questions, applying plagiarism checker and so on). Therefore, my decision is "Major Revision."
