# Supplementary material for: Exploring the behavioral determinants of COVID-19 vaccine acceptance among an urban population in Bangladesh: Implications for behavior change interventions
Source: PLoS One. 2021 Aug 23;16(8):e0256496. doi: 10.1371/journal.pone.0256496 (PMC8382171; doi:10.1371/journal.pone.0256496)
Supplement: Attachment — Submitted filename: Response to reviewers comments_R2.docx [file pone.0256496.s007.docx]

July 28, 2021

Dear Academic Editor,

We are pleased to provide a revised manuscript entitled: **Exploring the Behavioral Determinants of COVID-19 Vaccine Acceptance among an Urban Population in Bangladesh: Implications for Behavior Change Interventions.** In this round, we do not have specific comments from the respected reviewers and they declared that we have addressed their comments in previous round. The only comment from the academic editor was on the data availability, which has been already uploaded in the system during revision round 1. However, we have made corrections on the manuscript, mostly on grammatical issues and choice of words – as part of proof-reading. This was done by a native English speaker who is also a co-author.

We look forward to the review of our revised manuscript and hope that it is now considered acceptable for publication in PLoS One.

Sincerely,

Md. Abul Kalam.

**Specific responses to academic editor and reviewers:**

Thank you for submitting your manuscript to PLOS ONE. After careful consideration, we feel that it has merit but does not fully meet PLOS ONE’s publication criteria as it currently stands. Therefore, we invite you to submit a revised version of the manuscript that addresses the points raised during the review process.

**Response:** Thank you so much for considering our manuscript for publication. We have updated the draft based on suggestions and recommendations.

**Comment:** ***Concerning Data Availability, you have declared that*** all data are fully available without restriction

Before accepting your article, you have to upload your data.

**Response:** Thank you so much for your concern. We uploaded the data file named **“S2 File: Tabulation sheet with data.”** This file can be found under the **"File Inventory"** tab.

**Comment:** If you would like to make changes to your financial disclosure, please include your updated statement in your cover letter. Guidelines for resubmitting your figure files are available below the reviewer comments at the end of this letter.

**Response:** Thanks so much for your suggestion. The financial disclosure is final as it was during the first submission.

**Comment:** If applicable, we recommend that you deposit your laboratory protocols in [protocols.io](http://protocols.io/) to enhance the reproducibility of your results. Protocols.io assigns your protocol its own identifier (DOI) so that it can be cited independently in the future. For instructions see: <http://journals.plos.org/plosone/s/submission-guidelines#loc-laboratory-protocols>. Additionally, PLOS ONE offers an option for publishing peer-reviewed Lab Protocol articles, which describe protocols hosted on [protocols.io](http://protocols.io/). Read more information on sharing protocols at <https://plos.org/protocols?utm_medium=editorial-email&utm_source=authorletters&utm_campaign=protocols>.

**Response:** Thanks so much for your suggestion. This is Not Applicable for us.

Journal Requirements:

Please review your reference list to ensure that it is complete and correct. If you have cited papers that have been retracted, please include the rationale for doing so in the manuscript text, or remove these references and replace them with relevant current references. Any changes to the reference list should be mentioned in the rebuttal letter that accompanies your revised manuscript. If you need to cite a retracted article, indicate the article’s retracted status in the References list and also include a citation and full reference for the retraction notice.

**Response:** Thanks so much for your suggestion. We have replaced one reference (no. 12) that was published in the national newspaper which is now available online.

[Note: HTML markup is below. Please do not edit.]

Reviewers' comments:

Reviewer's Responses to Questions

**Comments to the Author**

1. If the authors have adequately addressed your comments raised in a previous round of review and you feel that this manuscript is now acceptable for publication, you may indicate that here to bypass the “Comments to the Author” section, enter your conflict-of-interest statement in the “Confidential to Editor” section, and submit your "Accept" recommendation.

Reviewer #3: All comments have been addressed

Reviewer #5: All comments have been addressed

2. Is the manuscript technically sound, and do the data support the conclusions?

The manuscript must describe a technically sound piece of scientific research with data that supports the conclusions. Experiments must have been conducted rigorously, with appropriate controls, replication, and sample sizes. The conclusions must be drawn appropriately based on the data presented.

Reviewer #3: Yes

Reviewer #5: Yes

3. Has the statistical analysis been performed appropriately and rigorously?

Reviewer #3: Yes

Reviewer #5: Yes

4. Have the authors made all data underlying the findings in their manuscript fully available?

The [PLOS Data policy](http://www.plosone.org/static/policies.action#sharing) requires authors to make all data underlying the findings described in their manuscript fully available without restriction, with rare exception (please refer to the Data Availability Statement in the manuscript PDF file). The data should be provided as part of the manuscript or its supporting information, or deposited to a public repository. For example, in addition to summary statistics, the data points behind means, medians and variance measures should be available. If there are restrictions on publicly sharing data—e.g. participant privacy or use of data from a third party—those must be specified.

Reviewer #3: Yes

Reviewer #5: Yes

5. Is the manuscript presented in an intelligible fashion and written in standard English?

PLOS ONE does not copyedit accepted manuscripts, so the language in submitted articles must be clear, correct, and unambiguous. Any typographical or grammatical errors should be corrected at revision, so please note any specific errors here.

Reviewer #3: Yes

Reviewer #5: Yes

6. Review Comments to the Author

Please use the space provided to explain your answers to the questions above. You may also include additional comments for the author, including concerns about dual publication, research ethics, or publication ethics. (Please upload your review as an attachment if it exceeds 20,000 characters)

Reviewer #3: (No Response)

Reviewer #5: Thank you for addressing my concerns. I am now pleased to support this paper's publication in PLOS One.

7. PLOS authors have the option to publish the peer review history of their article ([what does this mean?](https://journals.plos.org/plosone/s/editorial-and-peer-review-process#loc-peer-review-history)). If published, this will include your full peer review and any attached files.


If you choose “no”, your identity will remain anonymous but your review may still be made public.


**Do you want your identity to be public for this peer review?** For information about this choice, including consent withdrawal, please see our [Privacy Policy](https://www.plos.org/privacy-policy).

Reviewer #3: No

Reviewer #5: No

**Comment:** [NOTE: If reviewer comments were submitted as an attachment file, they will be attached to this email and accessible via the submission site. Please log into your account, locate the manuscript record, and check for the action link "View Attachments". If this link does not appear, there are no attachment files.]

**Response:** Thanks so much for your suggestion. We addressed these comments during the revision Round 1.

**Comment:** While revising your submission, please upload your figure files to the Preflight Analysis and Conversion Engine (PACE) digital diagnostic tool, <https://pacev2.apexcovantage.com/>. PACE helps ensure that figures meet PLOS requirements. To use PACE, you must first register as a user. Registration is free. Then, login and navigate to the UPLOAD tab, where you will find detailed instructions on how to use the tool. If you encounter any issues or have any questions when using PACE, please email PLOS at [figures@plos.org](mailto:figures@plos.org). Please note that Supporting Information files do not need this step.

**Response:** Thanks so much for your suggestion. We have one figure which was formatted by PACE and uploaded during Revision round 1. The figure can be found in the File Inventory Tab.
